# The Avine Origin of Diphtheria

**Published:** 1888-06

**Authors:** 


### THE AVINE ORIGIN OF DIPHTHERIA.

It has been known for some years that birds and poultry are subject to a disease which corresponds to what in the human being is known as diphtheria. Several foreign observers have gone a step further, and have endeavored to show that the disease is capable of transmission from animals to human beings. Last year Dr. Turner drew up an interesting report for the local government board bearing on this alleged transmissibility, and he adduced a large number of observations which seemed to indicate a connection between a diphtheritic affection, not only in fowls, but in rabbits and cats, and a similar affection in man. The report comprised several instances in which the "strangles" in horses appeared to give rise to a like train of symptoms. In a thesis by Dr. Menziès, the transmission of the disease from animals to man is attributed to the dejections of the former. Diphtheritic affections among fowls are very common in Italy, and he quotes an instance in which four out of the five children of a medical man were attacked and died. In this case he incriminates the thatched roof, which was inhabited by colonies of fowls, geese, pigeons, etc. The dejections of these animals, washed off by the rain, found their way into the cistern or well from which the supply of drinking water was drawn. The danger had been recognized, and the servants were forbidden to use the water for domestic purposes. As the proper well was at some distance from the house, however, the order had been contravened, and when the epidemic appeared among the poultry, the children promptly succumbed. The remaining child owed his immunity to the fact that he never drank water. The same concatenation of circumstances was observed in several outbreaks elsewhere.

In 1884 an epidemic of diphtheria occurred in Skiatos, an island off the coast of Greece. Dr. Bild was called to a girl of twelve with symptoms of diphtheria, of which she died. On the day of her death seven other children in the same quarter of the town were attacked, but there was no reason to suppose that they had contracted it from the first. Subsequently the epidemic spread all over the town; it lasted five months, and caused thirty-six deaths. On inquiry it was ascertained that in the garden of the house at which the disease first manifested itself, a number of turkeys had succumbed to a disease which was characterized by the presence of false membranes of a gray color in the larynx. Moreover, one of the few surviving animals was found to be suffering from paralysis of the legs. The prevailing wind passed directly over the field, and blew the dust into the houses, thus affording every opportunity for the transmission of the virus. There would thus seem to exist *à priori* grounds for believing that diphtheria is mutually exchangeable between man and certain animals. It is therefore incumbent on all medical men, especially those who take an interest in their profession, to inquire into the history of all cases of diphtheria with a view to furnishing evidence bearing on this point. It is essentially a question to be decided by observation, and this only medical men can properly carry out.—*Medical Press and Circular*.

